# Mnemonic function in small vessel disease and associations with white matter tract microstructure^☆^

**DOI:** 10.1016/j.neuropsychologia.2017.07.027

**Published:** 2017-09

**Authors:** Athanasia Metoki, Rebecca L. Brookes, Eva Zeestraten, Andrew J. Lawrence, Robin G. Morris, Thomas R. Barrick, Hugh S. Markus, Rebecca A. Charlton

**Affiliations:** a Department of Psychology, Goldsmiths University of London, New Cross, London SE14 6NW, United Kingdom; b Department of Clinical Neurosciences, University of Cambridge, Cambridge CB2 1TN, United Kingdom; c Institute of Cardiovascular and Cell Sciences, St George's University of London, Cranmer Terrace, London SW17 0RE, United Kingdom; d King's College Institute of Psychiatry, Psychology & Neuroscience, London SE5 8AF, United Kingdom

**Keywords:** Long-term memory, Working memory, Cingulum, Uncinate, Ageing, Small vessel disease

## Abstract

Cerebral small vessel disease (SVD) is associated with deficits in working memory, with a relative sparing of long-term memory; function may be influenced by white matter microstructure. Working and long-term memory were examined in 106 patients with SVD and 35 healthy controls. Microstructure was measured in the uncinate fasciculi and cingula. Working memory was more impaired than long-term memory in SVD, but both abilities were reduced compared to controls. Regression analyses found that having SVD explained the variance in memory functions, with additional variance explained by the cingula (working memory) and uncinate (long-term memory). Performance can be explained in terms of integrity loss in specific white matter tract associated with mnemonic functions.

## Highlights

• Working and long-term memory are both impaired in SVD compared to healthy adults.
• SVD explained a significant proportion of the variance in both mnemonic functions.
• Variance in working memory was explained by microstructure of the cingula.
• Variance in long-term memory was explained by microstructure of the uncinate.

## 1 Introduction

Cerebral small vessel disease (SVD) refers to pathological processes that affect the cerebral arterioles, venules, and capillaries resulting in damage to the deep grey matter and white matter tissue (Wardlaw et al., 2013). In SVD, white matter damage reduces the efficiency of grey matter connections, disrupting the neural networks that support cognitive abilities (Lawrence et al., 2014, Shim et al., 2015). Particularly affected in SVD is executive function ability which includes mental flexibility and the ability to monitor performance. There is also impairment in working memory and this contrasts with relative sparing of long-term memory abilities (Brookes et al., 2012). (Please note we are using the definition of episodic long-term memory as described by Tulving, and distinct from working memory (Tulving, 1983)). These mnemonic functions rely on networks across the brain with strong connections to frontal regions (Metzler-Baddeley et al., 2011, Zahr et al., 2009). This paper will focus on white matter tracts that connect frontal to other brain areas and examine whether the relatively spared mnemonic function of long-term memory is associated with the uncinate fasciculi (white matter tracts connecting frontal and temporal lobes). The temporal lobes are often spared white matter damage in spontaneous SVD. This will be contrasted with the mnemonic function of working memory which relies on fronto-parietal connections often disrupted by white matter damage in the centrum semiovale, and often impaired in SVD.

Previous studies in neurotypical adults have demonstrated that working and long-term memory have both shared and unique cognitive features (Moscovitch et al., 2005). Working memory combines both executive and mnemonic function, the cognitive operations associated with it requiring maintenance of information, simultaneous processing and updating (Baddeley, 2012). Long-term memory involves memory for personally experienced events, remembered over a longer period (Kramer et al., 2003). Both abilities require information to be maintained in memory but working memory is more reliant on executive processes, particularly abilities that coordinate central processing resources to manipulate information or maintain it in an active state (Baddeley, 2012). In keeping with these shared and unique cognitive systems, brain networks also demonstrate shared and unique white matter involvement (Charlton et al., 2013). In a tract-based spatial statistic (TBSS) study in typical ageing, the white matter microstructure of the genu of the corpus callosum was associated with both mnemonic abilities, whereas working memory was additionally supported by the cingula and long-term memory by the uncinate fasciculi (Charlton et al., 2013).

Damage in SVD is focused on the white matter particularly in the centrum semiovale and deep grey matter nuclei, with relative sparing of white matter in the temporal lobes and cortical grey matter (Wardlaw et al., 2013, Shim et al., 2015). The location of such damage is likely to disrupt direct communication pathways between distinct brain regions, and thus be vital to information transfer (Reijmer et al., 2016). Working memory may be particularly affected in SVD due to both the location of damage in the brain, and because performance requires reiterative integration of information across multiple brain regions (Wardlaw et al., 2013, Charlton et al., 2008, O'Sullivan et al., 2004). While executive functions are generally considered to rely more on the prefrontal cortex, working memory has been shown to be supported by fronto-parietal interactions (Ranganath et al., 2003). Thus small amounts of damage to these regions and disruption of important white matter tracts (i.e. the cingula) that support working memory networks may have a disproportionate effect on cognition (Charlton et al., 2010a). Executive function impairments have been frequently described in SVD (Brookes et al., 2012, Brookes et al., 2015, Prins et al., 2005), although working memory has not been examined as regularly, poorer performance has been demonstrated (O'Sullivan et al., 2005). Although other studies have examined brain connectivity in SVD (Reijmer et al., 2016), to our knowledge no previous study has directly compared working and long-term memory in SVD with tracts of hypothesised importance.

Whilst long-term memory is generally thought to be relatively spared in the earlier stages of cognitive impairment associated with SVD (O'Sullivan et al., 2005, Schmidtke and Hüll, 2002), studies have demonstrated impairments in performance in sporadic (Prins et al., 2005, Cummings, 1994) and genetic forms of the disease (Buffon et al., 2006). In a population study with follow-up over five years, performance on a list-learning task declined but was not associated with change in white matter hyperintensities or cortical/subcortical atrophy (Prins et al., 2005). Long-term memory has been shown to rely on integration of information across multiple brain regions, including the prefrontal cortex and temporal lobe (Moscovitch et al., 2005, Daselaar et al., 2003). Previous studies have demonstrated that the microstructure of the uncinate fasciculi (connecting frontal and temporal lobes) is associated with long-term memory performance among health older adults and in disease states such as Alzheimer's disease (Bucur et al., 2008, Charlton et al., 2010b, Yasmin et al., 2008). The location of the uncinate fasciculi may mean that it is relatively spared in SVD, as damage in the temporal lobe is not common in spontaneous SVD (although such damage may occur in a genetic form of SVD, namely Cerebral Autosomal-Dominant Arteriopathy with Subcortical Infarcts and Leukoencephalopathy). In contrast, the cingula are located in the sensitive centrum semiovale region (Wardlaw et al., 2013, Charlton et al., 2010a). As yet it is unclear whether long-term memory ability (which is relatively spared in SVD), is associated with the microstructure of the uncinate fasciculi.

Previous studies have demonstrated strong associations between multiple areas of white matter and white matter tracts, with both working and long-term memory (Metzler-Baddeley et al., 2011, Zahr et al., 2009). Two tracts were selected for examination in this study based on their involvement in mnemonic functions and location in areas either typically affected (cingulum) or spared (uncinate) in spontaneous SVD (Wardlaw et al., 2013, Shim et al., 2015, Reijmer et al., 2016). Tracts were selected to be associated with hypothesised functions (see Hypotheses below), and to act as “control” tracts for the alternative function. Working memory relies on fronto-parietal connections and has been associated with the cingulum (Zahr et al., 2009, Charlton et al., 2013, Charlton et al., 2010a) which passes through the centrum semiovale. Long-term memory function is associated with microstructure of the uncinate connecting frontal and temporal regions with temporal regions generally being spared in sporadic SVD (Metzler-Baddeley et al., 2011, Charlton et al., 2014). Although the fornix is strongly associated with mnemonic function in both healthy ageing and disease (Metzler-Baddeley et al., 2011, Zahr et al., 2009), it is unclear if this tract is spared or damaged in SVD and does not connect directly to the frontal lobes, therefore was not considered in this analysis.

In this study we examine the associations between the specific mnemonic functions of working and long-term memory and the microstructure of the two white matter tracts (the cingula and uncinate fasciculi) hypothesised to support these functions, in a group of patients with SVD and healthy older adults. We hypothesise that 1) working memory ability will be lower in SVD compared to healthy older adults, whereas long-term memory will be relatively spared, 2) working memory performance will be associated with the microstructure of the cingula, whereas long-term memory performance will be associated with the microstructure of the uncinate fasciculi. Thus in Hypothesis 2, long-term memory will act as a “control” function for the working memory analysis and vice versa, and the uncinate will act as a “control” tract for the cingulum and vice versa.

## 2 Materials and methods

Ethical approval for this study was obtained from the Wandsworth REC and St George's local research ethics committee. All participants gave informed, written consent.

### 2.1 Participants

#### 2.1.1 SVD patients

A sample of 121 patients with SVD were recruited to the St George's Cognition and Neuroimaging in Stroke (SCANS) study from stroke services at three South London hospitals (St George's Hospital, St Thomas's Hospital, and King's College Hospital). SVD was defined as a clinical lacunar stroke syndrome (Bamford et al., 1991), with confluent leukoaraiosis (Fazekas Scale, grade 2 or more) on MRI (Fazekas et al., 1987), and with an anatomically appropriate lacunar infarct on MRI. SCANS is a longitudinal study assessing both cognition and brain changes using MRI. Data presented in this paper utilise only baseline data. Baseline MRI scanning and cognitive testing was performed at least three months after any stroke to avoid the influence of acute ischaemia on cognition or MRI. All participating patients were fluent in English. Exclusion criteria in the SCANS study were: any cause of stroke or leukoaraiosis other than SVD, cortical infarcts of any size, sub-cortical infarcts > 1.5 cm, evidence of large artery disease (vertebral, carotid, or intracranial stenosis), any other source of cardiac embolism, major psychiatric disorders (with the exception of depression which was not an exclusion variable), and any other major central neurological system disorders. Participants with contraindications to MRI scanning, including claustrophobia, were also excluded. From the sample of 121 participants, a further 15 were excluded (3 due to missing cognitive measures; 12 due to poor quality images in the DTI sequence and inability to extract one or more white matter tract). The final sample population used for this study was 106 SVD patients.

#### 2.1.2 Healthy older adults

A stroke-free control group was used for comparison with the SVD MRI data. Forty healthy older adults (HOA) were recruited from the four-year follow-up of the longitudinal GENIE (St George's Neuropsychology and Imaging in the Elderly) study (Charlton et al., 2006). GENIE includes neuropsychological assessments and MRI completed at baseline and repeated after 2 and 4 years. Data from the participants at the 4 year follow-up are included in this analysis as MRI acquisition used the same MR scanner as the SCANS project. For full details of the study see (Charlton et al., 2006). In brief, participants were recruited through family doctor lists, were aged 50–90 at baseline, had English as a first language, and were free from any neurological or psychiatric problems. All participants included in this analysis remained cognitively intact with abilities within the normal range and had no evidence of neurological problems. Of the 40 participants, five had incomplete MRI, so the final sample was 35 HOA. The final sample (n = 141) used in this study included 106 SVD patients and 35 HOA controls.

### 2.2 Neuropsychological assessments

In both studies participants completed a battery of standardised neuropsychological tests, administered by a trained research assistant. Working memory was measured using forward and backwards span from the Digit Span Test (DST) of the Wechsler Memory Scale – Third edition (WMS-III) (Wechsler et al., 1998). Long-term memory was measured using Immediate Recall (LM1) and Delayed Recall (LM2) on the Logical Memory subtest of the WMS-III (Wechsler et al., 1998). Raw scores were transformed into z-scores based on the mean and standard deviation of the healthy control group, and a mean of the z-scores for LM1 and LM2 was used to describe long-term memory. A comparison of the age-scaled scores for LM1 and LM2 with DST in the HOA group revealed no significant differences in performance (LM1: t = −.144, p = .886; LM2: t = 1.33, p = .192), therefore it was deemed appropriate to use the HOA means and standard deviations to produce z-scores for the SVD group.

The Mini-Mental State Examination (MMSE) was administered as a screen for general cognitive impairment (Folstein et al., 1975). Eleven individuals in the SVD group and no healthy older adults scored ≤ 24 on the MMSE, the cut-off for risk for dementia. Analyses were performed including and excluding these “at risk” individuals.

### 2.3 Image acquisition

MRI scanning was performed on a General Electric 1.5 T Signa HDxt MRI scanner equipped with magnetic field gradients of up to 33 mTm^−1^ (GE Electric, Milwaukee, WI). A proprietary head coil was used for transmission and reception. Diffusion-weighted images (DWI) were acquired axially using a diffusion-sensitized spin-echo planar imaging (EPI) sequence (TE = 93.4 ms, TR = 15,600 ms, FOV = 240 × 240 mm^2^, acquisition matrix = 96 × 96) across fifty-five 2.5 mm thick slices providing whole brain coverage and isotropic voxels (i.e. 2.5 mm × 2.5 mm × 2.5 mm voxel resolution). Four images without diffusion sensitization (b = 0 s mm^−2^) were acquired followed by DWI (b = 1000 s mm^−2^) in 25 non-collinear directions that were equally spaced on the sphere in positive and negative gradient directions.

### 2.4 Image analysis

Images were simultaneously subject movement and eddy current distortion corrected using the FSL Eddy Tool (EDDY, FMRI Software Library, FSL version 5.0; FMRIB Analysis Group, Oxford, UK, http://www.fmrib.ox.ac.uk/fsl (Andersson, 2016)). TrackVis version 0.6.1 and Diffusion Toolkit version 0.6.4 (www.trackvis.org/dtk/) were used to analyse the DWI data. Each participant's DWI data was uploaded to Diffusion Toolkit. Diffusion Toolkit computed DTI, FA and MD maps from the DWI data output from the subject movement and eddy current correction step. For each participant, deterministic tractography was performed in native space on the whole brain, by initiating tracking at each voxel centre using interpolated streamlines computed using a step length of .25 mm; tractography was terminated when the angle threshold was greater than 45° or FA fell below .15. Whole-brain fiber tracts were generated and imported into TrackVis for visualisation and analysis.

**Fig. 1:**
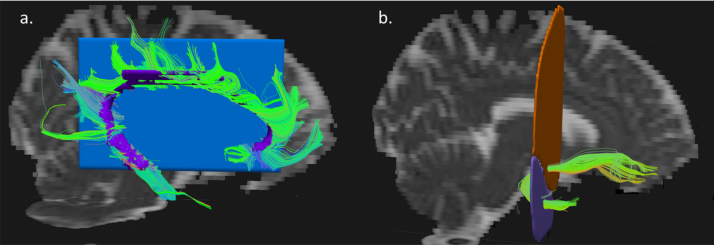
Example regions of interest used in the extraction of the (a) cingulum showing the inclusion region of interest (purple) and mid-sagittal exclusion region of interest (blue) (b) Uncinate fasciculus fibers were extracted between frontal (orange) and temporal (purple) regions of interest. The extracted fibers are shown on mean diffusivity images. (For interpretation of the references to color in this figure legend, the reader is referred to the web version of this article.).

**Fig. 2:**
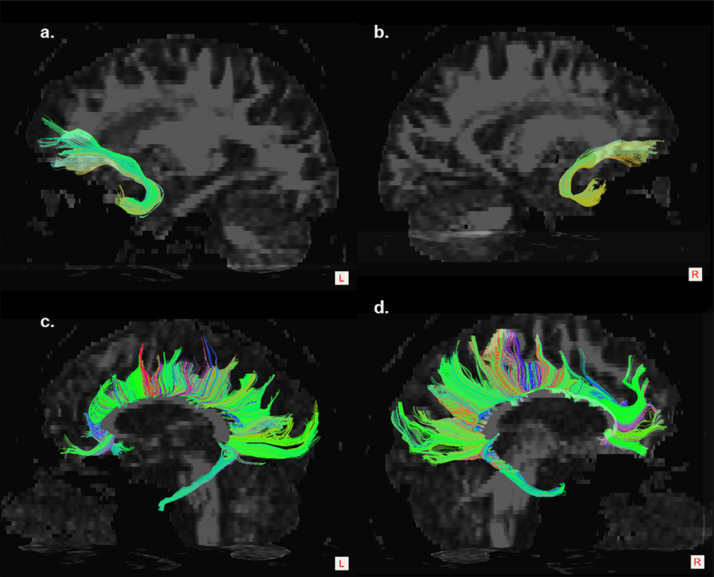
Examples of extracted tracts: a) left and b) right uncinate fasciculi; c) left and d) right cingula.

To extract the cingula and uncinate fasciculi, hand-drawn regions of interest (ROI) were delineated on direction encoded colour FA maps and fibers passing between ROIs (for the uncinate fasciculi) and through the ROI (for the cingula) were retained. The cingula were initially extracted using a single cigar shaped ROI placed over the most dorsal fibers of each cingulum (as identified from the direction encoded FA map) with an anterior-posterior course, as described in the method of Catani and Thiebaut de Schotten (Catani and Thiebaut de Schotten, 2008). To be able to accurately extract the entire cingulum this ROI was extended into the anterior and posterior branches of the cingulum to identify all cingulum fibers in the frontal and temporal lobes for all subjects. A further mid-sagittal exclusion ROI was required to prevent fibers passing across the corpus callosum (see Fig. 1a). The uncinate fasciculi were extracted using two ROIs as described in the method of Wakana et al. Wakana et al. (2007). The first ROI was placed over the frontal lobe in the most posterior coronal slice that separates the temporal and frontal lobe; a second ROI was drawn over the temporal lobe in the most dorsal axial slice that separates the two lobes (see Fig. 1b). TrackVis was used to calculate mean FA values within each tract of interest, and FA values were used in the analysis as a measure of white matter tract microstructure. See Fig. 2 for further examples of a participant's extracted bilateral cingulum and uncinate fasciculi.

### 2.5 Statistical analysis

Statistical analysis was performed using IBM SPSS Statistics, version 22 (IBM Corp, 2013). ANOVA and ANCOVA (controlling for education) examined differences between groups on cognitive variables and FA values in white matter tracts. Holm-Bonferroni statistics were applied to correct for multiple comparisons. A paired *t*-test examined differences in mnemonic performance within the SVD group. Pearson's correlational analyses were used to examine the associations between cognitive function and white matter tract integrity. Stepwise linear regression analyses were performed in turn to examine variables that explained variance in working and long-term memory. Independent variables entered in a single block were group, and FA of tracts of interest (left and right uncinate fasciculi, left and right cingula). Regression analyses were repeated including education as an additional independent variable and excluding individuals with MMSE scores ≤24.

## 3 Results

### 3.1 Group differences

SVD and HOA groups did not differ on measures of age or gender distribution, but group differences were noted for education level and MMSE (see Table 1). ANOVA demonstrated significantly lower scores for individuals with SVD compared to healthy older adults on both working and long-term memory scores. ANCOVA was performed to explore group differences in memory scores controlling for education level. Group differences on working (F = 21.40, p < .001) and long-term memory (F=6.46, p = .012) remained significant, after controlling for education level. The pattern of results remained unchanged when individuals scoring ≤ 24 on the MMSE were excluded from the analysis (see Table 1).

**Table 1:** Mean and standard deviations for demographic and cognitive data, and FA and MD tract values.

	**SVD (n = 106)**	**Healthy older adults (n = 35)**	**Group differences**	**Group differences excluding ≤ 24 on MMSE**
Age	69.52 (9.75)	69.63 (9.29)	F = .003, p = .954	F = .082, p = .776
Sex (m,f)	70,36	22,13	X^2^ = .117, p = .732	X^2^ = .001, p = .975
Highest education level	3.14 (2.37)	5.37 (2.17)	F = 24.28, p < .001 ***	F = 20.42, p < .001 ***
	Range = 1–9	Range = 1–9		
MMSE	27.74 (2.40)	29.11 (1.13)	F = 10.76, p = .001 ***	–
MMSE excluding scores ≤24	28.38 (1.37)	29.11 (1.13)	–	F = 8.05, p = .005 **
Long-term memory (z-score)	−1.06 (1.27)	0 (1)	F = 20.19, p < .001 ***	F = 15.06, p < .001 ***
Working memory (z-score)	−1.28 (1.06)	0 (1)	F = 39.80, p < .001 ***	F = 33.77, p < .001 ***
FA Right Uncincate	.3479 (.0252)	.3563 (.0261)	F = 2.79, p = .097	F = 2.09, p = .151
FA Left Uncinate	.3631 (.0219)	.3704 (.0255)	F = 2.62, p = .108	F = 1.64, p = .203
FA Right Cingulum	.3757 (.0232)	.3795 (.0374)	F = .466, p = .496	F = .125, p = .724
FA Left Cingulum	.3576 (.0207)	.3637 (.0365)	F = 1.43, p = .234	F = .770, p = .382
MD Right Uncincate	.00086643 (.000050)	.00082437 (.000050)	F = 17.98, p < .001 ***	F = 16.58, p < .001 ***
MD Left Uncinate	.00085750 (.000044)	.00082430 (.000053)	F = 13.22, p < .001 ***	F = 11.65, p = .001 ***
MD Right Cingulum	.00081821 (.000043)	.00080110 (.000065)	F = 2.99, p = .086	F = 1.75, p = .188
MD Left Cingulum	.00080120 (.000042)	.00078451 (.000071)	F = 2.71, p = .103	F = 1.59, p = .210

* p < 05; ** p < .01; *** p < .001; ± not significant after multiple comparison correction.

### 3.2 SVD differences in memory performance

A paired-sample *t*-test was performed to assess whether there were significant differences in performance on working and long-term memory tasks in the SVD group. Individuals with SVD show poorer performance for working memory (mean z-score = −1.28, sd = 1.06) compared to long-term memory (mean z-score = −1.06, sd = 1.27) tasks, although this did not reach significance (t = −1.97, p = .051). When this analysis was repeated excluding those with MMSE scores ≤ 24, the results show the same pattern of poorer performance on working memory (mean z-score = −1.19, sd = 1.05) compared to long-term memory (mean z-score = −.873, sd = 1.19) tasks, and this difference was significant (t = −2.64, p = .010). Analyses were not performed for the HOA group, as z-scores were calculated using this data.

### 3.3 Correlations

Working memory correlated significantly with FA and MD values in all white matter tracts (see Table 2). Long-term memory correlated significantly with MD in all tracts, but only with left hemisphere tracts for FA values. After controlling for multiple comparisons, working memory remained significantly correlated with FA in right and left uncinated fasciuli, right cingulum, and MD in the right uncincate fasciculus; long-term memory remained significantly correlated with MD in the right cingulum. After excluding individuals with ≤ 24 on the MMSE, correlations between working memory and FA values remained significant although they were reduced in magnitude and did not remain significant after multiple comparison correction (see Table 2). Associations between long-term memory and FA values no longer reached significance.

**Table 2:** Correlations between long-term and working memory and FA and MD tract values.

	**Whole sample (n = 141)**		**Sample excluding individuals ≤ 24 on MMSE (n = 130)**	
	**Long-term Memory**	**Working Memory**	**Long-term Memory**	**Working Memory**
FA Right Uncincate	r = .130, p = .129	r = .268, p = .001 ***	r = .161, p = .065	r = .225, p = .010 *±
FA Left Uncinate	r = .212, p = .013 *±	r = .255, p = .003 **	r = .157, p = .075	r = .196, p = .025 *±
FA Right Cingulum	r = .165, p = .055	r = .242, p = .005 **	r = .137, p = .120	r = .210, p = .016 *±
FA Left Cingulum	r = .207, p = .015 *±	r = .216, p = .011 *±	r = .126, p = .153	r = .172, p = .050 *±
MD Right Uncincate	r = −.202, p = .018 *±	r = −.252, p = .003 *	r = −.148, p = .097	r = −.207, p = .019 *±
MD Left Uncinate	r = −.180, p = .034 *±	r = −.176, p = .039 *±	r = −.116, p = .195	r = −.130, p = .146
MD Right Cingulum	r = −.237, p = .005 **	r = −.260, p = .002 **	r = −.145, p = .108	r = −.176, p = .050 *±
MD Left Cingulum	r = −.207, p = .015 *±	r = −.193, p = .024 *±	r = −.112, p = .210	r = −.102, p = .257

* p < 05; ** p < .01; *** p < .001; ± Not significant after multiple comparison correction.

### 3.4 Regression analyses

#### 3.4.1 Working memory

The final model explained 23.7% of the variance, (F(2,138) = 20.06, p < .001). Variables that significantly contributed to the model were group which explained 18.1% of the variance (β = −.404, p < .001) and the mean FA of the right cingulum which explained an additional 5.6% of the variance (β = .237, p = .003).

For the regression analysis including education as an independent variable and excluding individuals with MMSE scores ≤ 24, the model was significant and explained 29% of the variance (F(3,126) = 16.05, p < .001). Education level (17.4%, β = .295, p = .001), group (7.4%, β = −.284,p = .001) and right cingulum FA contributed to the model (4.2%, β = .205, p = .010).

#### 3.4.2 Long-term memory

The final model explained 14.9% of the variance long-term memory (F(2138) = 11.28, p < .001). Group explained 12.3% of the variance (β = −.331 p < .001), and FA of the left uncinate fasciculus explained a further 2.6% of the variance (β = .163, p = .048).

The analysis was repeated including education as an independent variable and excluding individuals with MMSE scores ≤ 24. The model explained 22.4% of the variance (F(2,127) = 17.13, p < .001) and included education level (19.6%, β = .375, p < .001) and group (2.7%, β = −.178, p = .044).

Regression analyses were performed using MD values as the predictor variables. For working memory the model was significant and the same variables were included in the regression mode (F(2,138) = 19.02, p < .001). Group explained 18.1% of the variance (β = −.384 p < .001) and MD in the right cingulum explained an additional 4.6% of the variance (β = −.219 p = .006). For long-term memory, the model was significant (F(2,138) = 11.55, p < .001) but explained by group (12.3%, β = .317, p < .001) and MD of the right cingulum (2.9%, β = −.175, p = .036).

#### 3.4.3 Comparison analyses

In order to assure that tract specific microstructure was not simply reflecting whole brain microstructure, the above regression analyses were repeated including (in turn) a whole brain FA measure and a measure of white matter hyperintensities. Results of the regression analyses remained unchanged. Methods and results for these analyses are described in full in the Supplementary materials.

## 4 Conclusions

The aim of this study was to examine the specific associations between different aspects of mnemonic function and white matter tracts of hypothesised importance in SVD. Our results demonstrate that both working and long-term memory are impaired in SVD compared to age-matched controls, and that mnemonic functions are associated with white matter tracts. The SVD group demonstrated poorer performance than age matched HOA, on both measures of working memory and long-term memory. Although difficulties in working memory performance have been previously reported and were expected due to known executive function difficulties in SVD (O'Sullivan et al., 2005), long-term memory difficulties are less commonly described (Prins et al., 2005). Long-term memory decline has been described in SVD in a longitudinal study but was not associated with structural brain changes (Prins et al., 2005). In the current study, individuals with SVD demonstrate lower performance on working and long-term memory measures compared to age-matched HOA when education level was included as a covariate and individuals with more severe cognitive decline (≤ 24 on MMSE) were excluded. The results suggest that while working memory is significantly more impaired than long-term memory in SVD, long-term memory performance is still significantly lower in SVD than in healthy aged peers.

When regression analyses were performed to explain mnemonic performance in the whole sample, performance was explained by presence of SVD and by tracts specific to each mnemonic function. SVD group membership explained 18.1% of the variance in working memory and 12.3% of the variance in long-term memory. This suggests that the disease has an impact on both mnemonic functions, but a relatively greater impact on working memory performance. Working memory may be at greater risk of impairment in SVD due to its reliance on iterative neural networks that utilise white matter tracts through the centrum semiovale, an area at particular risk in SVD (Wardlaw et al., 2013, Charlton et al., 2010a).

In keeping with centrum semiovale tracts being affected in SVD and with previous findings in healthy ageing, variance in working memory performance was also explained by the integrity of the cingulum using either FA or MD values in the regression models (Charlton et al., 2013, Kennedy and Raz, 2009a). In previous studies of typical ageing the cingula have been shown to underlie working memory performance through white matter in frontal and parietal regions as well as in fronto-parietal tracts (Kennedy and Raz, 2009b). In contrast, a small but significant amount of the variance in long-term memory performance was explained by FA in the left uncinate fasciculus, although this tract did not contribute to the model after controlling for education and excluding those with low cognitive ability (MMSE ≤ 24). Furthermore when MD values were used in the regression model, group and MD of the right cingulum significantly explained the variance in long-term memory performance. Results in the whole sample are in keeping with previous studies which have demonstrated that both temporal and frontal regions support long-term episode memory performance (Henson and Gagnepain, 2010), as well as temporal white matter and fronto-temporal connections (Kennedy and Raz, 2009b, Davis et al., 2009).

The regression analyses in combination with the correlational analyses suggest a complex pattern of associations between mnemonic function and white matter tracts. Correlational analyses reveal that both working and long-term memory correlate significantly with bilateral cingula and uncinate fasciculi, although the magnitude of correlations differs between mnemonic functions and white matter tract. These results are in keeping with previous studies in healthy adults that have shown working and long-term memory to be supported by complex networks of grey and white matter in frontal, temporal and parietal regions (Moscovitch et al., 2005, Charlton et al., 2013, Ranganath et al., 2003, Daselaar et al., 2003). This same pattern of brain-mnemonic function associations is observed here in SVD patients, although the magnitude of associations is reduced when excluding individuals with impaired cognitive function (i.e. MMSE ≤ 24).

The study has a number of limitations. Although the SVD sample size is moderate the HOA group is small and represents individuals who have remained in a longitudinal study over a four-year period. However, robust differences in cognitive function are noted between the groups, even when analyses control for differences in educational level, and many analyses are performed on the SVD group alone. In this study we have extracted specific tracts of interest that are hypothesised to be associated with mnemonic function; however examination of further white matter tracts or whole brain analyses may provide additional information about the pattern of associations across a wider brain network. In this study, tractography ROIs were manually drawn on FA maps but the ROIs were identified to be as large and inclusive as possible to reliably identify the tracts of interest and to provide minimal bias to the computed tract FA. The limitations of DTI may potentially affect the ability of deterministic tractography to obtain the most accurate extraction of the cingulum and uncinate fasciculus. Application of a correction for cerebrospinal fluid (CSF) contamination (Metzler-Baddeley et al., 2012) could potentially improve the reliability of fiber tracking through regions of low FA, such as for tracts close to CSF spaces or that pass through regions of white matter lesions that are present in SVD. In this study, the cingulum may be affected by CSF-contamination as portions of the pathway will pass through regions containing white matter lesions in the centrum semiovale. Furthermore, deterministic or probabilistic tractography of the cingulum bundle may be affected by regions of white matter fiber crossing between projection and association fibers that DTI tractography techniques cannot fully resolve (Farquharson et al., 2013). In this study this effect may manifest as a reduction of measured FA in fiber crossing regions of the cingulum. The use of constrained spherical deconvolution based tractography (Tournier et al., 2004, Tournier et al., 2007) methods could potentially overcome some of these effects (Farquharson et al., 2013) but would have limited applicability to the current DTI data set as it was acquired at 1.5 T with a relatively low number of diffusion directions at a b-value of 1000 s mm^−2^. Ideally acquisition of DWI with high angular diffusion gradient direction resolution, high voxel resolution and at 3 T or higher would enable more accurate reconstruction of fiber anatomy using spherical deconvolution based tractography techniques. Finally, mnemonic functions were measured using two standardised neuropsychological assessments. Although it could be argued that a broader range of assessments may better describe the profile of mnemonic function, these measures are reliable and robust and have yielded significant results in the current analyses.

While executive function difficulties are widely acknowledged in SVD there has been less focus on mnemonic function with relatively few studies specifically examining working memory (O'Sullivan et al., 2005). Long-term memory is seldom examined as deficits are less severe than other cognitive difficulties in SVD, and long-term memory is not as impaired in SVD as in disorders such as Alzheimer's disease. In this study we demonstrate that both working and long-term memory are impaired in SVD compared to HOA, and are associated with the microstructure of white matter tracts. A better understanding of mnemonic difficulties in SVD may impact patient care, by both acknowledging subtle difficulties and tailoring information to reduce working and long-term memory load.
