# Supplementary material for: Mnemonic function in small vessel disease and associations with white matter tract microstructure
Source: Neuropsychologia. 2017 Sep;104:1–7. doi: 10.1016/j.neuropsychologia.2017.07.027 (PMC5637155; doi:10.1016/j.neuropsychologia.2017.07.027)
Supplement: Supplementary file 1 — Supplementary material [file mmc1.docx]

**Supplementary Material**

Additional analyses were performed to examine the role of whole brain fractional anisotropy (FA) and white matter hyperintensities (WMH) on mnemonic function. As the results did not yield any significant findings these analyses were not included in the paper, but are reproduced here for reference.

**General Methods**

*MRI Acquisition*

Images were acquired on a 1.5-T General Electric Signa HDxt MRI system (General Electric, Milwaukee, WI, USA) with a maximum gradient amplitude of 33 mTm^-1^ and a proprietary head coil. Sequences were acquired across the whole brain. Participants were positioned in the head coil in a neutral position with an alignment marker at the nasal bridge to standardise head position; foam pads and a Velcro strap were used to minimise head movement. T1-weighted acquisition: Coronal spoiled gradient recalled echo T1-weighted (SPGR) sequence-TR/TE = 11.5/5 ms, FOV= 240x240 mm^2^, matrix = 256x192, Flip Angle = 18⁰, 176 contiguous slices of 1.1 mm thickness. Axial Fluid Attenuated Inversion Recovery (FLAIR) sequence-TR/TE/TI = 9000/130/2200 ms, field of view (FOV) = 240x240 mm^2^, matrix= 2566192, 28 contiguous slices of 5 mm thickness. See main paper for DTI acquisition details.

**Whole Brain Analyses**

*Methods*

Whole brain analyses have been previously reported (Lawrence et al 2013). In brief, FA maps were calculated from the diffusion tensor, fit using DTIFIT in FMRIB’s Diffusion Toolkit (FDT; FSL v4.1; Smith et al 2004). Anatomical T1-weighted images were then coregistered to the diffusion volumes using FMRIB’s Linear Registration Tool (FLIRT; FSL v4.1; Jenkinson & Smith 2001) and the resulting affine transformation used to bring a whole brain ROI into alignment with the FA map. We then calculated for each subject the mean average FA value within brain voxels.

*Results*

*Correlations*: While whole brain Mean FA is associated with working memory performance in the SVD group (r=.241, p=.014; likely reflecting the white matter damage common in SVD as well as the network to support working memory function); it is not associated with working memory performance in the HOA group (r=.119, p=.510). Whole brain mean FA is also not associated with LTM in either group (SVD: r=.147, p=.138; HOA: r=.165, p=.359).

*Regression Analyses*: Stepwise regression analysis including a whole brain FA variable (alongside the tracts of interest) was performed. The final models remained unchanged, i.e. the tracts and group remain significant predictors of each mnemonic function and whole brain FA is excluded from the model (LTM model, Whole Brain Mean FA, Beta=.061, p=.578; WM model, Whole Brain Mean FA, Beta=.129, p=.185).

**White Matter Hyperintensities** (WMH)

*Methods*

White matter hyperintense regions of FLAIR images were delineated by a single rater, using the semi-automated DISPUNC program (Grimaud et al 1996; David Plummer, University College London, UK). Lesions that were >2 mm in diameter were delineated. Whole brain lesion maps were generated and lesion load calculated as the percentage of parenchymal brain volume (described in full by Lawrence et al 2013). This lesion load quantity was subjected to a variance stabilising transformation (log_10_) before use as a predictor in regression models.

*Results*

*Regression Analyses*: We repeated the stepwise regression analysis including WMH volume, however the models remained unchanged. For each mnemonic function the tracts and group variables significantly explained the variance but WMH was excluded from the model. For interest the beta weights for WMH when it is excluded from the regressions models are as follows: LTM model, WMH, Beta=.013, p=.882; WM model, WMH, Beta=-.059, p=.463.

**References**

Grimaud, J., M. Lai, J. Thorpe, P. Adeleine, L. Wang, G. J. Barker, D. L. Plummer, P. S. Tofts, W. I. McDonald, and D. H. Miller. 1996. ‘Quantification of MRI Lesion Load in Multiple Sclerosis: A Comparison of Three Computer-Assisted Techniques.’ *Magnetic Resonance Imaging* 14 (5): 495–505.

Jenkinson, M., and S. Smith. 2001. ‘A Global Optimisation Method for Robust Affine Registration of Brain Images.’ *Medical Image Analysis* 5 (2): 143–156.

Lawrence, Andrew J., Bhavini Patel, Robin G. Morris, Andrew D. MacKinnon, Philip M. Rich, Thomas R. Barrick, and Hugh S. Markus. 2013. ‘Mechanisms of Cognitive Impairment in Cerebral Small Vessel Disease: Multimodal MRI Results from the St George’s Cognition and Neuroimaging in Stroke (SCANS) Study.’ *PLoS One* 8 (4): e61014.

Smith, Stephen M., Mark Jenkinson, Mark W. Woolrich, Christian F. Beckmann, Timothy E J. Behrens, Heidi Johansen-Berg, Peter R. Bannister, et al. 2004. ‘Advances in Functional and Structural MR Image Analysis and Implementation as FSL.’ *NeuroImage* 23 Suppl 1: S208–S219.
